# The Practical Application of the Individual Care Plan for Pediatric Palliative Care: A Mixed-Method Study

**DOI:** 10.3390/children11080967

**Published:** 2024-08-11

**Authors:** Chantal Y. Joren, Judith L. Aris-Meijer, Leontien C. M. Kremer, Suzanne C. Hofman, Hester Rippen-Wagner, Ria Slingerland-Blom, Chantal van der Velden, Meggi A. Schuiling-Otten, Project Team ICP, A. A. Eduard Verhagen, Marijke C. Kars

**Affiliations:** 1 University of Groningen, University Medical Center Groningen, Beatrix Children’s Hospital, Hanzeplein 1, 9713 GZ Groningen, The Netherlands; 2 Princess Maxima Centre for Pediatric Oncology, Heidelberglaaan 25, 3584 CS Utrecht, The Netherlands; 3 University Medical Center Utrecht, Wilhelmina Children’s Hospital, Lundlaan 6, 3584 EA Utrecht, The Netherlands; 4 Dutch Foundation Child and Hospital, 3527 GV Utrecht, The Netherlands; 5 Dutch Centre of Expertise in Children’s Palliative Care, Mercatorlaan 1200, 3528 BL Utrecht, The Netherlands; 6 University Medical Center Groningen, Beatrix Children’s Hospital, Hanzeplein 1, 9713 GZ Groningen, The Netherlands; 7 Center of Expertise in Palliative Care Utrecht, Julius Center of Health and Primary Care, UMC Utrecht, Universiteitsweg 100, 3584 CG Utrecht, The Netherlands

**Keywords:** pediatric palliative care, individual care plan, pediatric advanced care planning, shared decision-making

## Abstract

Background/Objective: The Individual Care Plan (ICP) for pediatric palliative care was developed to provide person-centered care for the individual child and family. Currently, a lack of clarity remains regarding the use and function of the ICP in daily practice. To further implement the ICP, it is important to identify how parents and healthcare professionals use the ICP and which obstacles or benefits are experienced. Methods: This mixed-method study used qualitative interviews and quantitative questionnaires in (bereaved) parents and healthcare professionals with experience with the ICP. Results: Parents and healthcare professionals used the ICP to establish a joint plan for care and treatment of the child to coordinate care and to achieve child- and family-centered care. This includes both obstacles that complicate achieving care goals and benefits that make it easier. Furthermore, responsibilities for the ICP remained unclear, and there was no set point in the illness trajectory for drawing up the ICP. Conclusions: Parents and healthcare professionals use the ICP as intended. However, uncertainties regarding timing, roles and responsibilities prevent optimal use of the ICP. Agreements on timing and responsibilities are needed for further ICP implementation in daily pediatric palliative care practice.

## 1. Introduction

In 2015, the Individual Care Plan (ICP) for pediatric palliative care (PPC) was developed by translating the general guideline recommendations ‘palliative care for children’ into an individualized care plan [1]. The aim of the ICP was to provide high-quality PPC and person-centered care for the individual child with a life-limiting or life-threatening illness and their family [1]. The ICP could be used to document and share the preferences, desires and agreements regarding the current and future care and treatment of the child. The subgoal was to thereby support coordination and continuity of the PPC. The ICP has been implemented nationwide, is considered as ‘good practice’, is recommended for use in the revised guideline ‘palliative care for children’ [2], and is in use by all eight Children’s Palliative Care (CPC) teams in the Netherlands.

A first small-scale pilot study directly following the development of the ICP in 2015 showed promising results for the ICP: its content covered all elements of PPC making it a comprehensive document and healthcare professionals (HCPs) evaluated it as an improvement of the situation at the time [1]. PPC is quite challenging due to the diverse care trajectories, involvement of numerous HCPs, and the movement of the child between care organizations and home [3,4,5]. This poses risks for HCPs operating in isolation, unaware of agreements made between parents and other HCPs, challenging the coordination and continuity of care [3,4,5]. Thus the ICP was seen as a tool to improve this situation.

Despite the intention to use the ICP for all children with a life-limiting or life-threatening illness and the nationwide implementation, the ICP is currently used for a limited number of children. Furthermore, HCPs indicated that even though the ICP is regarded as a helpful tool, it is also perceived as difficult to work with. However, it remains unclear what this means, and literature on the topic is scarce. Therefore, we evaluated the use of the ICP from parents’ and HCPs’ experiences, the obstacles or benefits it presents, and which agreements, if any, are necessary, thereby ensuring the implementation of the ICP.

## 2. Methods

### 2.1. Design

A cross-sectional mixed-method methodology with a convergent parallel design was used, fitting with a pragmatic theoretical approach [6]. The study is part of a larger multi-phase study of which a detailed description is provided elsewhere [7]. We used qualitative research encompassing a thematic analysis of individual interviews with parents and focus group interviews with HCPs and a questionnaire among parents and HCPs. Data used in this paper were collected between April and August 2021.

### 2.2. Sample

The inclusion criteria for the qualitative and quantitative study were identical. Eligible participants were bereaved and non-bereaved parents whose children had an ICP that was created within the past three years, as well as HCPs with practical experience with the ICP in PPC [7].

### 2.3. Recruitment

#### 2.3.1. Recruitment of Parents

The Children’s Palliative Care (CPC) teams emailed eligible parents, inviting them to take part in the study through an individual interview and/or questionnaire. Parents were able to self-enroll via the project website. In addition, a call for participation with a URL to the questionnaire was posted on the social media channels of the Dutch Foundation Child and Hospital and Dutch Centre of Expertise in Children’s Palliative Care.

#### 2.3.2. Recruitment of HCPs

To recruit eligible HCPs for the questionnaire, CPC teams and network coordinators sent out invitation emails containing the URL to the online questionnaire to all eligible HCPs, along with a call to invite other HCPs involved in ICP’s. Moreover, a call for participation was posted on the social media channels of the Dutch Foundation Child and Hospital and Dutch Centre of Expertise in Children’s Palliative Care.

For the recruitment of HCPs for the focus group interviews the CPC teams and network coordinators identified all HCPs with prior ICP experience. In selecting HCPs, variation was sought with respect to regions, functions, lines of care and disciplines. Invitations were sent by the CPC teams or network coordinators to the selected HCPs. HCPs could enroll for a focus group interview via the project website.

### 2.4. Data Collection and Measurements

#### 2.4.1. Individual and Focus Group Interviews

We used videocalled semi-structured individual and focus group interviews to explore the experiences of parents and HCPs with ICP in daily practice. The topic guides for the individual and focus group interviews, created from the literature and the research team’s knowledge, covered procedures, goals, the role and contribution of child/parents, and how they are reflected in the ICP; see Supplements S1, S2 and S3. The topic guide for the focus group interviews was tested by interviewing two HCPs. To ensure understanding of both working with and drawing up an ICP, two types of focus groups were used: (1) HCPs with experience with working with the ICP and (2) HCPs with experience with drawing up the ICP.

#### 2.4.2. Online Questionnaires

The questionnaires built on the questionnaire from the ICP pilot study [1], with additional questions on the background variables, content and process of the ICP, guided by implementation aspects [8]; see Supplements S4 and S5.

### 2.5. Data Analysis

#### 2.5.1. Qualitative

All interviews were videotaped, transcribed verbatim and pseudonymized. After transcription, the videotapes were deleted. Thematic analysis was used to gain insight into the experiences of parents and HCPs with the ICP in daily practice [9,10]. The qualitative analysis was conducted by a team of three researchers (CJ, JA, MK) using open and axial coding. To become familiar with the data, two individual parent interviews and one focus group interview with HCPs were individually read and coded by CJ, JA and MK. The codes and interpretations of the researchers were then compared and discussed until consensus was reached, resulting in a preliminary code tree. Based on this code tree, all transcripts were coded by CJ. The codes were evaluated by the team and the content of codes and the process of categorization were adjusted during the coding process. To guarantee constant comparison, the team went back and forth between the different steps. As such, the team worked towards the identification of the primary categories and corresponding codes. The analysis was facilitated by the software program Atlas ti 9.

#### 2.5.2. Quantitative

The quantitative data were descriptively analyzed in SPSS version 28 using mean, ±SD, median and interquartile range.

#### 2.5.3. Mixed-Method Integration and Analysis

The qualitative and quantitative data were analyzed separately to confirm the convergent parallel design. After analysis the results were integrated and compared to assess for confirmation, expansion or discordance [11]. Confirmation occurred if the qualitative and quantitative findings each confirmed the results of the other [11,12]. Expansion occurred when the findings from these two data types differed, thereby enhancing understanding of the use of the ICP [11,12]. Discordance occurred if the two types of showed discrepancies, contradictions or disagreements [11,12]. A narrative approach was used to describe the results [11].

#### 2.5.4. Validity and Reliability

Multiple strategies were used to ensure validity and reliability [13]: first, by making use of data triangulation, meaning that we explored the perceptions of different groups of participants (parents and HCPs from different disciplines). Second, researcher triangulation was used. The analysis was performed by three researchers who worked together to develop the code tree, where consensus was important. Third, the interviews were recorded and transcribed verbatim, which enhances validity and reliability. Fourth, a summary of the results was discussed with parents from the parent panel consisting of five parents who had experience with the ICP and HCPs from the project team enhancing the validity of our results.

## 3. Results

### 3.1. Respondents

Twenty-seven parents completed the questionnaire, of which nine also participated in an individual interview which lasted approximately an hour (Table 1). One parent couple participated in the questionnaire. In total, 161 HCPs participated, of whom 16 participated in both a focus group interview and questionnaire, 24 in the focus group interviews only and 153 in the questionnaire only (Table 2). In total, there were four focus group interviews lasting 1.5 h each with up to six HCPs.

### 3.2. Use of the ICP in Daily Practice

Parents and HCPs used the ICP (a) to establish a joint plan for care and treatment of the child and (b) to achieve child- and family-centered care. These goals include both obstacles that make achieving the goals more difficult and benefits that make it easier. In addition, obstacles and benefits were found considering (c) the responsibilities surrounding the ICP and (d) the timing of drawing up the ICP. See Table 3 for the complete overview.

- To establish a joint plan for care and treatment of the child

Parents and HCPs both used the ICP to coordinate care by collaboratively establishing a joint plan for current and future care and treatment of the child, aiming to achieve interdisciplinary collaboration with all HCPs involved across lines of care. Mostly CPC team nurses took the lead in drawing up an ICP, together with the primary treating physician and parents. If the CPC team wasn’t involved, an ICP was often not drawn up. Parents felt heard by HCPs in the process of drawing up an ICP; they felt like integral members of the team, believed that their role as parents mattered and that they had a say in the decisions made. *“I think it was more in the conversations and the feeling that you were heard… At least we really felt heard as parents. […]. Well we experienced it as support. So not the fact that a doctor decides what will happen. They have always given their opinions as a doctor; we would do that, we wouldn’t do that or whatever you want now, that’s okay, that’s possible.”(Parent #7).* To draw up the ICP, HCPs felt it is necessary to gain insight into the wishes and needs of the child and parents and to discuss current and future care, including treatment limitations and end-of-life care. Parents and HCPs both considered these conversations extremely valuable, even more so than the actual document. Nevertheless, often the ICP itself facilitated the initiation of these conversations. Some HCPs discussed various scenarios for the disease trajectory with parents, acknowledging that care preferences can vary across scenarios. *“[..] It is important to discuss the scenarios with parents; what do we expect regarding how the child may pass away? And that it can be acute. Or that it could be with pneumonia. Or that it could be slow or cardiac arrest. And it’s crucial to outline these possibilities to some extent. This often brings clarity. And that we also have different scenarios. So that there are different wishes and needs in terms of treatment instructions and especially the limitations therein. And I’ve noticed that that is just incredibly helpful. So whether or not to resuscitate is not very black and white; but in one situation yes and in another situation no. And from a vision, the parents’ life philosophy, norms and values.” (Nurse, hospital, FG drawing up ICP, #2)*

Parents used the ICP to share the joint plan for care and treatment with HCPs involved to ensure continuity of care and to prevent having to repeat and explain everything in an already difficult period. *“[…], because what I just like is that everything is clear and transparent to everyone when [the child] is admitted to the hospital.”(Parent, #2).* Furthermore, parents appreciated being able to read back the discussed and agreed-upon information. especially since it was sometimes a lot of information to remember. HCPs also used the ICP for the continuity of care. Primary care HCPs used the ICP as a reference to draw up their organization specific care plan. They appreciated being able to pre-read and know what had been discussed and agreed upon, before meeting the child and family. Furthermore, by collaboratively establishing an ICP, care could be effectively provided across lines of care, since the possibilities and limitations offered by the different locations/settings were taken into account. *“And I think that’s exactly what you should use it for. Because what we often notice is that certain agreements are made by the hospital, such as: yes, you have to do it so and so. While people have absolutely no idea how things are going at home. […] You know, so if you can discuss that in advance, especially if a child is not terminal, but does have a life-limiting illness, I think that should only have added value.” (Nurse, home care, FG working with ICP, #19).*

b. To achieve child- and family-centered care

HCPs wish to provide child- and family-centered care through an ICP, by exploring the wishes and needs of child and parents, often in multiple conversations. These conversations were sometimes described as ‘difficult’ by HCPs because they entailed discussing treatment limitations, end-of-life care and futile medical care. Although HCPs were eager to provide child- and family-centered care, conflicts arose when parents requested everything to be done for their child and HCPs believed it was medically irresponsible to do so. These conflicts became even more pronounced with drawing up an ICP, as agreements could not be made and therefore not written down. *“If, as a doctor, you simply don’t think it’s responsible what parents want, then you don’t come to a shared decision. Yeah, well, I think the conclusion was that you just have to keep talking, talking, talking, but you can never really go against it. But yes, those are the abrasive aspects of our profession, which are simply very painful.”(Nurse, hospital, FG drawing up ICP, #2).* Through the ICP, all involved HCPs were aware of the wishes and needs of the child and/or parents, which made it possible to adapt care accordingly. This enabled the provisions of appropriate care regardless of the location, ensuring continuity of care. *“But what is nice sometimes is that you do indeed know what has already been discussed with parents for example […] And there have been many conversations with the mother, but we found out through the ICP that the mother does not want the child to hear that he is dying. That you think: oh yes, those are things that suddenly come to light through the ICP.” (Other, home care, FG working with ICP, #18)*.

In the interviews, parents emphasized the importance of maintaining control in the care of their child. Having an ICP offered parents a sense of reassurance, ensuring them that a plan was in place and that everything was well-coordinated. This was substantiated in the questionnaire where 60% (n = 15) of the parents said the ICP helped them to have more control in the care of their child. The ICP provided them with a feeling of security, assuring them that undesired events could be averted. *“It sounds very strange, but for us, it was very necessary to clarify that things that we did not want to happen would not happen either.” (Parent, #3).* HCPs also observed that having an ICP offered parents reassurance, offered them guidance and contributed to their process. *“While I believe that, for parents, and I have noticed this in the past years, it can really be helpful to gain peace in the process or in their minds; “it’s spinning for us, and we don’t know [what to do]”. And if you have such a plan, it becomes much clearer for them.” (Nurse, hospital, FG drawing up ICP, #16).*

Children were generally not involved in drawing up an ICP. This was also shown in the questionnaire, as most parents (n = 19, 90.5%) and HCPs (n = 58, 95.1%) considered it not possible to involve the child due to developmental delay, communications difficulties or too-young age. In these cases, parents used the ICP to convey the child’s voice. ensuring that even in their absence, the voice of the child was heard and the wishes of parents regarding the care of their child were met, thereby ensuring quality of care. *“It’s not about us, it’s about [the child] in the end. So even though [the child] can’t talk and we have to be [the childs’] voice, it’s all about [the child]. So yes, it is important that it [the ICP] is indeed with [the child].” (Parent #2).*

In the questionnaire, almost three-quarters of the parents (n = 19) agreed that the agreements in the ICP and the goals of care and treatment as stated in the ICP were in line with what they considered important for their child and family. Of the HCPs, 80.3% (n = 102) said the agreements in the ICP were in line with what the child and parents considered important for themselves and the family.

c. The responsibilities surrounding the ICP

Primary care HCPs believed hospital HCPs were responsible for drawing up an ICP, but they themselves could recognize the need for an ICP and reach out to the hospital. HCPs found it important that the HCP that drew up the ICP was experienced in PPC, knew what the consequences of medical decisions would be and was able to guide parents in the decision making process. It was often unclear for HCPs who was responsible for drawing up, sharing and keeping the ICP up-to-date. HCPs pressed the need for someone to be responsible to prevent mistakes. Not knowing who was responsible seemed to obstruct HCPs from relying fully on the information written in the ICP. Nurses felt they could take the coordinating role in ICPs, but they seemed hesitant to fully position themselves that way.

In the questionnaire, HCPs were asked who they felt was responsible for the drawn-up ICP. Half said the primary treating physician is responsible, 19.5% said the person who drew-up the ICP is responsible and 26.6% said other, i.e., the care team, CPC team, and joint responsibility of primary treating physician, parents and CPC team (Table 4). In the interviews, all HCPs felt the primary treating physician should be involved in the ICP. However, CPC team nurses often had difficulties involving the primary treating physician as they were often hesitant to draw up an ICP. This complicated the ICP process. *“Well, that doctors are reluctant when I come up with a plan [ICP]. Maybe the plan itself, but also the idea of making the plan.” (Nurse, hospital, FG drawing up ICP, #16).*

CPC team nurses and pediatricians felt responsible for guiding others, mainly general practitioners (GPs) and home care nurses, to ensure continuity and quality of care. They saw it as their responsibility to educate GPs and home care nurses in palliative care through the ICP, because there was a fear that due to little or no expertise, wrong actions would be taken or mistakes would be made, which would prolong the suffering of the child. *“We ran into the fact that it simply wouldn’t work otherwise. Then things were done or things were started that were really not contributing to those children, but actually only prolonged the suffering.” (Nurse, hospital, FG drawing up ICP, #12).* They did not primarily draw up an ICP for themselves but rather to support parents and other HCPs in caring for the child. By providing them with step-by-step guidelines, mainly for the terminal phase, they hoped that GPs and home care nurses could prepare themselves.

d. The timing of drawing up the ICP

In the questionnaire, half of the parents (n = 13) indicated that the ICP for their child was drawn up more than two years after the onset of disease or diagnosis (Table 5). Almost half of the HCPs (41%, n = 53) said that the last ICPs they had drawn up were drawn up within 2 months after the manifestation of disease or diagnosis (Table 5). The interviews made apparent that there was no fixed time for drawing up an ICP, however most ICPs were drawn up close to the terminal phase, even though HCPs saw advantages in drawing up an ICP early in the course of illness. *“We work with ICPs, but mostly when children are palliatively terminal, that’s actually the main reason ICPs are used, I have to say, in our experience. In fact not in children who have a life-limiting disease.”(Nurse, home care, FG working with ICP, #19).* HCPs were sometimes reluctant to bring up the ICP early because they were afraid to confront parents with the end of their child’s life. Some parents sensed the reluctance on the part of HCPs to initiate conversations, and found it difficult having to request the ICP themselves. Parents felt that the initiative should have come from HCPs instead of from themselves. *“[…] I have the idea that we are actually often the ones who are one step ahead or… At a certain point we indeed had a conversation with the pediatrician and the neurologist, and I thought, I think the neurologist actually finds it more difficult to talk about it than we do.” (Parent, #8).*

The timing of drawing up an ICP also influenced the use of the ICP in practice. When the ICP was drawn up early in the course of illness, the ICP was mostly used as a reference instrument. However, during the terminal phase, the ICP was both used as a reference instrument as well as a literal step-by-step guide to provide terminal care by both parents and HCPs. At certain times, the ICP was used more intensively, e.g., during hospitalization, periods of deterioration, or in the final stages of life.

In cases where the ICP was drawn up near the terminal phase of the child, both parents and HCPs felt the information within the ICP lagged behind reality, making it less effective in improving quality of care. Nevertheless, drawing up the ICP still facilitated connections among HCPs, ensuring interdisciplinary collaboration. As a result, the final phase proceeded more smoothly, thereby enhancing quality of care. *“Yes, and I think sooner. Look, with us it’s of course… We had a bad diagnosis for a long time, so there could have been a plan much sooner… Because in the last weeks, I found it really intense to go through with a nurse.” (Parent, #1).* Parents preferred drawing up an ICP when their child was in a clinically stable condition rather than during a crisis. This preference was shared by some HCPs, underscoring the significance of affording parents the chance to reflect on their preferences for their child’s care in advance, rather than in the midst of a crisis. *[…] Well, I don’t know if there are times when things are really going well for [the child], but in general it is of course not necessarily useful to discuss what you are not going to do next time, if you have been very close to that point. And you think, well, if we hadn’t done it then, then [the child] wouldn’t have been here anymore. So it’s easier to discuss things like that when you’re not just past that point.” (Parent #8).*

## 4. Discussion

This study provides a much-needed insight into the utilization of the ICP in daily PPC practice. It shows that parents and HCPs use the ICP to establish a joint plan for care and treatment of the child and to achieve child- and family-centered care. By doing so, they want to achieve coordination, continuity and quality of care. However, obstacles and benefits were found. CPC team nurses often took the lead in drawing up an ICP, and they mainly drew up ICPs to guide other HCPs. There was no fixed moment in the illness trajectory for drawing up the ICP and it remained unclear who was responsible for the drawn up ICP.

Parents in our study used the ICP, among other things, to voice their wishes regarding care and treatment of their child, so that also in their absence, care aligned with their wishes could be provided. In PPC, parents often find it difficult to entrust someone else with the care of their child [14]. They feel the need to coordinate care and remain hypervigilant when HCPs are unfamiliar with their child and when HCPs lack information regarding care and treatment, while they would rather spend time with their child [14,15]. Through the use of the ICP, information on care and treatment can be shared with HCPs safeguarding continuity and quality of care, which allows parents to feel reassured.

It was often unclear for HCPs who was responsible for drawing up, keeping up-to-date and sharing the ICP, complicating the use of the ICP in practice. Often, CPC team nurses took the lead in drawing up an ICP. In other studies, nurses were also the ones responsible for drawing up an ICP [16,17]. However, most of the eight CPC teams have only been in existence for a few years [18]. Currently, CPC teams lack the capacity to draw up an ICP for every child with a life-limiting or life-threatening disease, and many are not under their care. To ensure the use of the ICP, it is important to come to roles and responsibilities regarding the use of the ICP together with HCPs. The person drawing up the ICP should be competent in PPC and have regular contact with the child [19].

Remarkably, CPC team nurses and pediatricians did not draw up ICPs so much for themselves, but mainly for GPs and primary care HCPs. This arose particularly from their sense of responsibility for the quality of PPC, and they hoped to guide others with less experience in providing PPC by using the ICP. It is not surprising that they feel this responsibility, since guiding other HCPs falls within their duties [20]. However, ICPs have only been used for adult patient education and support before, and not for HCPs [21]. In the Netherlands, PPC is still rarely addressed in nursing and medical education [22]. It is therefore not unexpected that there is a need to share knowledge on PPC. The question, however, is whether the ICP is the right instrument for this. Further research should look into it.

There seems to be a shift occurring in the timing of drawing up an ICP. While most ICPs still seem to be drawn up when death is imminent, an increasing number of ICPs are now also being drawn up within 2 months after the manifestation of disease or diagnosis. However, it seems difficult to define the right time to initiate drawing up an ICP, if there even is a ‘right’ time [23]. Looking back, most bereaved parents in our study would have wanted an ICP earlier in the course of illness. However, HCPs were sometimes reluctant to initiate an ICP early in fear of confronting parents. Nonetheless, a previous study shows that parents value open and honest communication and for HCPs to not sugarcoat the information [24]. Furthermore, early initiation of PPC has shown to improve quality of life, promote physical and emotional well-being, and to decrease patient suffering [25]. We therefore advise to initiate an ICP early in the course of illness. However, future research should look into overcoming barriers for early initiation of PPC.

### Strengths and Limitations

This study was the first to evaluate the use of the Dutch ICP in daily PPC practice, providing us with valuable new information that can underlie improvement of the use and implementation of the ICP. By combining qualitative and quantitative methods and in including both parents and HCPs, we were able to build on the strengths of both methods, and provide a fuller picture of the ICP in daily practice. A possible limitation could be self-enrolment, which could have led to a selection of participants who were interested in improving the ICP. Furthermore, the study population consists of 90% women. However, this is not surprising since women make up 80% of the professionals in healthcare in the Netherlands [26]. We were not able to include children, which could have provided different insights into the use of an ICP in practice, as children can differ in their experiences from their parents. However, a large part of the population for whom an ICP was drawn up could not be included due to developmental delays, communication issues, too-young age or because they had passed away. The quantitative sample of parents is quite small; however, we were able to include both parents from living and deceased children with a wide range of diagnoses, thus providing a broad perspective. Conducting the individual and focus group interviews digitally made it possible to proceed during the COVID-19 pandemic without risking contamination. However, it was therefore necessary for participants to have access to a digital device with internet, which turned out to be no problem. Furthermore, conducting interviews online has proven to be as effective as face-to-face interviews [27].

## 5. Conclusions

Parents and HCPs use the ICP as intended: to provide person-centered care for the individual child and family, to thereby achieve coordination, continuity and quality of care. However, uncertainties regarding timing, roles and responsibilities prevent optimal use of the ICP. Agreements on timing and responsibilities should be made for further implementation of the ICP in daily PPC practice.

## Figures and Tables

**Table 1:** Characteristics of the parents respondent group.

	n *=* 27 ^a,b^ (n,%)
Age, mean (min–max)	44 (26–60)
Highest completed education	
Basic vocational education and training (EQF ^c^ = 2)	3 (11.1)
Secondary vocational qualification or higher general secondary education/pre-university education (EQF ^c^ = 4)	12 (44.4)
Bachelor’s degree (EQF ^c^ = 6)	12 (44.4)
Child’s main diagnosis	
Neurological	8 (30.8)
Cardiological	2 (7.7)
Metabolic	4 (15.4)
Oncological	12 (46.2)
Child alive/child deceased	
Alive	6 (25.9)
Deceased	20 (74.1)
Age at inclusion, mean (min–max)	10 (0–25)
Age at death, mean (min–max)	11 (0–19)

^a^ All parents who were interviewed also completed the questionnaire. ^b^ Sometimes n = 26 instead of n = 27, due to the participation of a parent couple in the questionnaire. ^c^ European Qualifications Framework.

**Table 2:** Characteristics of the healthcare professionals respondent group.

	Total (n = 161)	Questionnaire ^a^ (n = 153)	Focus Group Interviews ^b^ (n = 24)
Gender female/male	148/13	140/13	22/2
Age, mean (min–max)	NA	45 (23–64)	NA
CPC network			
Northeast	33	30	6
Southeast	14	13	2
Limburg/Brabant	19	19	4
Southwest	9	8	2
Holland-Rijnland	21	20	3
North-Holland & Flevoland	19	18	2
Utrecht	26	25	5
Region unknown	20	20	-
Profession			
Medical doctor	32	27	7
General practitioner	12	12	0
Nurse	90	87	16
Other	27	27	1
Setting			
Hospital	63	56	14
Primary care	98	97	10
Role ICP			
Draw up ICP	63	58	8
Working with, did not draw up ICP	97	95	8
Both	NA ^c^	NA ^c^	8

^a^ This also includes healthcare professionals who participated in the focus groups (n = 16). ^b^ This also includes healthcare professionals who have completed the questionnaire (n = 16). ^c^ In the questionnaire they were asked what role they generally have.

**Table 3:** Complete overview of obstacles and benefits.

Category	Benefits	Obstacles
**(a) To establish a joint plan for care and treatment of the child**	Collaboratively establishing a joint plan for current and future care and treatment	If a Children’s Palliative Care team was not involved an ICP ^a^ was often not drawn up
	Achieving interdisciplinary collaboration across lines of care	
	Ensuring coordination and continuity of care	
	Parents do not have to repeat everything every time	
	Parents feel like integral members of the team	
	Drawing up ICP initiates conversations on wishes and needs of child and parents and discussing current and future care and treatment	
	Discussing different possible treatment scenarios	
**(b) To achieve child- and family-centered care**	Through the ICP, HCPs ^b^ were aware of wishes and needs of child and parents, wherefore appropriate care could be provided regardless of location	“Difficult” conversations discussing treatment limitations, end-of-life care and futile medical care
	Even in parents’ absence, their wishes could be met through the use of the ICP	When agreements on care and treatment could not be made, nothing could be written down in the ICP
	Having an ICP gave parents a sense of reassurance that care was well-coordinated and undesired events could be averted	Often not possible to involve the child in drawing up an ICP
**(c) Responsibilities surrounding the ICP**	Used the ICP to educate others	Responsibilities for drawing up, sharing and keeping the ICP up-to-date were unclear
		Experience in PPC ^c^ is needed to draw up an ICP
**(d) Timing of drawing up the ICP**	ICP used as step-by-step plan and reference document	Fear to confront parents with child’s end-of-life
	Facilitates interdisciplinary collaboration	No fixed moment in illness trajectory for drawing up ICP

^a^ Individual Care Plan (ICP). ^b^ Healthcare Professionals (HCPs). ^c^ Pediatric Palliative Care (PPC).

**Table 4:** Who is responsible for the drawn up Individual Care Plan?

Who Is Responsible for the Drawn up Individual Care Plan?	Healthcare Professionals (n = 128), n (%)
Primary treating physician	69 (53.9)
Person who drew up the Individual Care Plan	25 (19.5)
Other	34 (26.6)

**Table 5:** Time between manifestation of disease/diagnosis and drawing up an Individual Care Plan.

Period between Manifestation of Disease/Diagnosis and Drawing up an Individual Care Plan	Parents (n = 26), n (%)	Healthcare Professionals (n = 130), n (%)
0–2 months	6 (23.1)	53 (40.8)
3–6 months	3 (11.5)	22 (16.9)
7–12 months	3 (11.5)	9 (6.9)
Longer than a year, but no longer than two years	1 (3.8)	6 (4.6)
Longer than two years	13 (50.0)	8 (6.2)
I don’t know	-	32 (24.6)

## Data Availability

The data presented in this study are available under request from the corresponding author and with permission from the principal investigator and co-investigators due to the rare conditions that children have who qualify for PPC, and therefore, their possibility to be traced. We therefore want to protect their privacy by controlling access to the data.

## Supplementary Materials

The following supporting information can be downloaded at: https://www.mdpi.com/article/10.3390/children11080967/s1, Supplement S1: Individual Care Plan Palliative Care for Children; Supplement S2: Topic guide parent; Supplement S3: Topic guide healthcare professionals; Supplement S4: Questionnaire parents; Supplement S5: Questionnaire healthcare professionals.

## Abbreviations

ICP	Individual Care Plan
PPC	Pediatric palliative care
HCPs	healthcare professionals
CPC	Children’s Palliative Care
GPs	General practitioners

## Appendix A. Project Team ICP

L. Beeloo ^F^, M.E. Bos ^H^, A.P. Groenenberg ^I^, M.T.J. Hermans-Peters ^J^, M. de Jong ^G^, C. Joosen ^K^, J. Verheijden ^F^, M. Vrolijk ^F^, M.M.J.P. Wielders ^L^.

^F^ Dutch Centre of Expertise in Children’s Palliative Care, Mercatorlaan 1200, 3528 BL Utrecht, The Netherlands

^G^ University Medical Center Groningen, Beatrix Children’s Hospital, Hanzeplein 1, 9713 GZ Groningen, The Netherlands

^H^ Leids University Medical Centre, Willem-Alexander Childrens Hospital, Albinusdreef 2, 2333 ZA Leiden, The Netherlands

^I^ Amsterdam University Medical Centre, Emma Childrens Hospital, Meibergdreef 9, 1105 AZ Amsterdam, The Netherlands

^J^ Radboud University Medical Centre, Amalia Childrens Hospital, Geert Grooteplein Zuid 10, 6525 GA Nijmegen, The Netherlands

^K^ Erasmus Medical Centre, Sophia Childrens Hospital, Wytemaweg 80, 3015 CN Rotterdam, The Netherlands

^L^ Maastricht University Medical Centre, MosaKids Childrens Hospital, P. Debyelaan 25, 6229 HX Maastricht, The Netherlands
