# Supplementary material for: The Practical Application of the Individual Care Plan for Pediatric Palliative Care: A Mixed-Method Study
Source: Children (Basel). 2024 Aug 11;11(8):967. doi: 10.3390/children11080967 (PMC11352542; doi:10.3390/children11080967)
Supplement: Supplementary file 1 [file children-11-00967-s001.zip › Supplementary_S1_ICP_1.0_DEF.pdf]

Individual Care Plan Palliative Care for Children

1. Care plan information

This care plan

- Is drawn up by: Name of the initial author
  Name(s) of other author(s)
- Version number: Enter a number
- Is drawn up on: Select a date
- Approved by the child (always for 12+): Choose an item.
  on: Select a date
- Approved by parents on: Select a date
- Approved by chief practitioner: Select a date
- To be revised on: Select a date ...

Description of what was discussed with parents/child (regarding prognosis, care goals etc...., will follow later):
Has everything been discussed (including prognosis)? In what words? What does the child know?

2. General information

Child's information

- Name Child's name
- Date of birth Date of birth
- Address Street, House number
  Postal code, city
- Second address (if applicable) Name / description (e.g., mother's address)
  Street, House number
  Postal code, city
- Phone numbers
  - Child Click here to enter text.
  - Father Click here to enter text.
  - Mother Click here to enter text.
- Email address Click here to enter text.
  Click here to enter text.
- Language spoken Choose item. ...
- Proficiency in the Dutch language Yes
- Calculation details
  - Weight Click here to enter text. kg on Select date
  - Hight Click here to enter text. cm on Select date

Medical situation

- Diagnosis Click here to enter text.

- Date of diagnosis Select date
- Severity / prognosis:
  Click here to enter text.
- Relevant medical history:
  Click here to enter text.
- Allergies Click here to enter text.

Care team

**24-hour Emergency number**		Name / Number	
**Chief practitioner**		Name, title	
Phone	Phone number	Email address	Email address
After-hours		Name / Number	
**Care coordinator**		Name, title	
Phone	Phone number	Email address	Email address
After-hours		Name / Number	
**Secondary practitioner /pediatrician**		Name, title	
Phone	Phone number	Email address	Email address
**Palliative care team coordinator**		Name, title	
Phone	Phone number	Email address	Email address
**General practitioner**		Name, title	
Phone	Phone number	Email address	Email address
After-hours		Name / Number	
**Primary responsible nurse unit**		Name, title	
Phone	Phone number	Email address	Email address
**Home care**		Name	
Primary contact person		Name, title	
Phone	Phone number	Email address	Email address
Other contacts		Name, title / Number	
After-hours		Name / Number	
**Pharmacy**		Name	
Phone	Phone number	Email address	Email address
Afther-hours		Name / Number	
**Pediatrician reginal hospital**		Name	
Phone	Phone number	Email address	Email address
After-hours		Name / Number	
**Psychologist**		Name, institute	
Phone	Phone number	Email address	Email address
**Medical social worker**		Name, institute	
Phone	Phone number	Email address	Email address
**Pedagogical Staff**		Name, institute	
Phone	Phonen umber	Email address	Email address

**Spiritual counselor**		Name, institute	
Phone	Phone number	Email address	Email address
**School / daycare**		Name, institute	
Phone	Phone number	Email address	Email address
**Other**		Name, institute	
Phone	Phone number	Email address	Email address
**Other**		Name, institute	
Phone	Phone number	Email address	Email address
**Other**		Name, institute	
Phone	Phonen umber	Email address	Email address

3. Social map / psychosocial aspects

Data

- *Family*
  - Father — Name, age, accupation
  - Mother — Name, age, occupation
  - Siblings — Names and ages of siblings
- *Living situation*
  Legal status, in case of separated partner(s), custody, and living arrangements
- *Housing conditions*
  Practical aspects (e.g., ground floor residence, location of bedroom and bathrooms)
- *Medical facilities at home*
  Description
- *Care support / Self-sufficiency*
  Degree of independence / level of assistance required
- *Daily routine*
  Description of daily routine
- *School / daycare*
  Description of school/daycare, coordination with school/daycare
- *Leisure activities (sports / hobby)*
  Brief description of sports/hobbies, names of associations, coordination
- *Social network of the child and family*
  Grandparents, neighbors, friend
- *Other*
  Other relevant aspects regarding the social map

Perception

- *Important aspects in the perception*
  What are the family’s wishes/goals/important aspects of perception
- *Religion/spirituality*
  What role does religion or spirituality play within this family?
- *Contact with psychosocial care providers*

Current contact with child psychologists

- *Relaxation exercises*

  Interventions that have a positive effect on fear/anxiety of the child
- *Other*

  Other aspects that are important with regard to perception

The manual contains extensive information on the psychosocial phases and aspects of palliative illness in the child, parents and environment.

Psychosocial aspects that require attention are:

Note here aspects as described in the manual that require special attention

Other comments/details regarding social map/psychosocial aspects

E.g. relevant psychosocial history of family member

4. Needs, wishes and goals

What are the child's personal wishes, expectations, needs and (general) goals?

Free entry

What are the personal wishes, expectations, needs and (general) goals of the parents?

Free entry

Agreements made about care goals based on expectations and wishes

Free entry

Any disputes/dilemmas

Note bottlenecks, for example where parents disagree with each other or with the clinician

Have agreements been made regarding treatment restrictions?

- Resuscitation policy Cleary record agreements
  - Circulation; drug support Choose an item. (explanation if needed)
  - Ventilation: suction Choose an item. (explanation if needed)
  - Ventilation: oxygen administration Choose an item. (explanation if needed)
  - Ventilation: mask and balloon Choose an item. (explanation if needed)
  - Ventilation: intubation/mechanical ventilation Choose an item. explanation if needed)
- Diagnostics Clearly record agreements
- Medication for infection (AB) Clearly record agreements
- Transfusion policy Clearly record agreements
- Admission Clearly record agreements
- Intensive care admission Clearly record agreements
- Fluid and nutrition Clearly record agreements
- Other: Clearly record agreements

End of life

Detail regarding end of life	Agreements/wishes (e.g. location)
Possible autopsy/donation	Discussed? Current wishes?
Farwell and funeral	Current wishes? Discussed with child?
Bereavement care	Agreements/wishes regarding bereavement care

5. Medication including dosage

Daily/weekly

<u>Overview</u>

Overview of medication

Ex.
XXX 3 daily xx mg
XXXX Once a week on Friday xx mg
XXXX 1 daily xx ug, iv.

<u>Timetable</u>

Time	Name	Form	Dose
…	…	…	…

If needed, currently:
Description of medication

Options to add, in case of…
Descripton of medication and when to give it

Present at the child/parents' home
What is available in the house? (think of future medication!) Both medication and material.

6. Nutrition

Wishes of parent/child regarding (tube)feeding : Click here if you want to enter text.

Description of feeding pattern
Description of daily nutrition/fluid intake/tube feeding)

Agreements regarding fluids, nutrition and supplements
Write down clearly

7. Symptomatology

Indicate which symptoms are currently relevant and which symptoms can be expected.

Symptoms - Current

Click here if you want to enter text.

Symptoms – Take into account possibly expected during diagnosis/prognosis
Click here if you want to enter text.

Symptoms – General wishes of parents/child
Click here if you want to enter text.

Symptoms – General comments
Click here if you want to enter text.

7a. Pain

Current situation: Click here if you want to enter text.

Expectation / "Is it likely that this will occur in this palliative process?":
Click here if you want to enter text.

Goals child/parents: Click here if you want to enter text.

Specific description of current situation: Pain scores, localizations, course

Diagnostics
Measuring instrument pain: Make your choice ...

Treatment - Medicinal
Nociceptive pain

! **Currently in step: 0 , any additions/adjustments:** Click here if you want to enter text. !

Step 1 – not opioids
Step 1a - Paracetamol (KF)
Dosage: Paracetamol Choose an item., Choose an item dd ... mg.
Administration form: e.g tablet
Step 1b – NSAIDs (KF ibu, KF diclo)
Dosage: Choose an item. Choose an item., Choose an item. dd ... mg.

Step 2 – Opioids for mild pain
Tramadol (KF)
Dosage: Step 1. Tramadol Choose an item., Choose an item. dd ... mg.
Step 2. Tramadol Choose an item., Choose an item. dd ... mg.

Step 3 – Opioids for severe pain
NB. **Avoid codeine, consider oxycodone, buprenorphine and PCA pump**
Morphine (KF)
Dosage:
Short-acting morphine Choose an item., Choose an item. ... mcg Kies een Choose an item.

Long-acting morphine Choose an item., Choose an item. ... mcg Choose an item..
Fentanyl patch: Click here if you want to enter text.
Rule for breakthrough pain: Choose an item.. ...
Schedule adjustment for long-acting opiate: Choose an item.. ...

Oxycodon (KF) of buprenorfine (FK)
Dosage: Choose an item. Choose an item., Choose an item. dd ... mg.

Neuropathic pain

NB. **Avoid phenytoin, carbamazepine and valproate, consider opioids**

Tricyclic antidepressant (FK)
Dosage: Amitriptyline Choose an item., Choose an item. dd ... mg.
Depending on the effect and side effects, gradually increase every 3-7 days if no effect yet.

Antiepileptic drugs (FK)
Dosage: Choose an item. Choose an item., Choose an item. dd ... mg.

Other pain (e.g. bone pain)

Other pain medication: Click here if you want to enter text.

Treatment – Non-medicinal
Actions taken: Click here if you want to enter text.

7b. Nausea/vomiting

Current situation: Click here if you want to enter text.

Expectation / "Is it likely that this will occur in this palliative process?":
Click here if you want to enter text.

Goals child/parents: Click here if you want to enter text.

Diagnostics
Keeping diary frequency/duration: Choose an item ...

Treatment – Non-medicinal
Actions taken: Click here if you want to enter text.

Treatment - Medicinal

Currently in step: 0 , any additions/adjustments: Click here if you want to enter text.

Step 1

Stap 1a - 5-HT3-receptor antagonist (KF ond)
Dosage: Ondansetron Choose an item., Choose an item. dd ... mg.
Stap 1b – D2-receptor antagonist (KF domp)
Dosage: Domperidon Choose an item., Choose an item. dd ... mg.

Further medication for nausea/vomiting (see manual)
Click here if you want to enter text.

7c. Constipation

Current situation: Click here if you want to enter text.

Expectation / "Is it likely that this will occur in this palliative process?":
Click here if you want to enter text.

Goals child/parents: Click here if you want to enter text.

Treatment- Non-medicinal
Actions taken: Click here if you want to enter text.

Treatment - Medicinal
Medication and dosage: Click here if you want to enter text.

7d. Dyspnea

Current situation: Click here if you want to enter text.

Expectation / "Is it likely that this will occur in this palliative process?":
Click here if you want to enter text.

Goals child/parents: Click here if you want to enter text.

Diagnostics
Measuring instrument dyspnea: Choose an item. ...

Treatment – Non-medicinal
Measures taken: E.g. in case of shortness of breath)2 for comfort, posture advice, etc.

Treatment - Medicinal

Dyspnea - *Morphine* (KF)
Dosage: Step 1. Morphine Choose an item., Choose an item. ... mcg Choose an item..
Step 2. Morphine Choose an item., Choose an item. ... mcg Choose an item..
Next steps: Click here if you want to enter text.

Restlessness/anxiety in dyspnea - *Benzodiazepines* (KF lora, KF mida), *as an addition to morphine*
Dosage: Choose an item. Choose an item., Choose an item. dd ... mg.

Other medication for dyspnea: Click here if you want to enter text.

7e-1. Cough

Current situation: Click here if you want to enter text.

Expectation / "Is it likely that this will occur in this palliative process?":
Click here if you want to enter text.

Goals child/parents: Click here if you want to enter text.

Comments on current situation
Click here if you want to enter text.

Treatment - Cause
Actions taken: Click here if you want to enter text.

Treatment- Non-medicinal

Actions taken: Click here if you want to enter text.

Treatment - Medicinal
Medication and dosage: Click here if you want to enter text.

7e-2. Rattling

Current situation: Click here if you want to enter text.

Expectation / "Is it likely that this will occur in this palliative process?":
Click here if you want to enter text.

Goals child/parents: Click here if you want to enter text.

Comments on current situation
Click here if you want to enter text.

Treatment – Non-medicinal
Actions taken: Click here if you want to enter text.

Treatment - Medicinal
Medication and dosage: Click here if you want to enter text.

7f. Fatigue

Current situation: Click here if you want to enter text.

Expectation / "Is it likely that this will occur in this palliative process?":
Click here if you want to enter text.

Goals child/parents: Click here if you want to enter text.

Comments on current situation
Click here if you want to enter text.

Diagnostics
Ever go through screening list? Choose an item. – Name screenings list

Treatment – Non-medicinal
Actions taken: Click here if you want to enter text.

Treatment - Medicinal
Medication and dosage: Click here if you want to enter text.

7g. Anxiety and depression

Current situation: Click here if you want to enter text.

Expectation / "Is it likely that this will occur in this palliative process?":
Click here if you want to enter text.

Goals child/parents: Click here if you want to enter text.

Comments on current situation
Click here if you want to enter text.

Diagnostics
Ever go through screening list? Choose an item. – Name screenings list
Possible other causes/triggers: Choose an item. - Click here if you want to enter text.

Treatment – Non-medicinal
Actions taken: Click here if you want to enter text.

Treatment – Medicinal
Medication and dosage: Click here if you want to enter text.

7h. Neurological symptoms

7h-1. Epilepsy

Current situation: Click here if you want to enter text.

Expectation / "Is it likely that this will occur in this palliative process?":
Click here if you want to enter text.

Goals child/parents: Click here if you want to enter text.

Comments on current situation
Click here if you want to enter text.

Treatment – Medicinal

- Attack medication (Attack duration of 5 minutes is t=0 for medicinal interventions)
  - Step 1, t=0, give Choose an item. Choose an item …mg one-off
    Step 2, t=5, give Choose an item. Choose an item. …mg one-off
    Insertion of IV desired by child/parents? Choose an item., possibly bone needle? Choose
  - Step 3, t=10, intravenous Choose an item. …mg
- Maintenance medication: Click here if you want to enter text.

7h-2. Movement disorders, spasticity, disability symptoms

Current situation: Click here if you want to enter text.

Expectation / "Is it likely that this will occur in this palliative process?":
Click here if you want to enter text.

Goals child/parents: Click here if you want to enter text.

Comments on current situation
Click here if you want to enter text.

Treatment – Non-medicinal
Actions taken: Click here if you want to enter text.

Treatment - Medicinal
Medication and dosage: Click here if you want to enter text.

7i. Hematological phenomena

Current situation: Click here if you want to enter text.

Expectation / "Is it likely that this will occur in this palliative process?":
Click here if you want to enter text.

Goals child/parents: Click here if you want to enter text.

Diagnostics
Last Hb: value mmol/l op date.
Trombo: value x 10^9/l op date.
other diagnostics: Click here if you want to enter text.

Treatment- Non-medicinal

History of transfusions: Describe previous transfusions + effect.
History of bleeding: Describe previous bleeding + measures taken + effect.
Emergency kit acute serious life-threating bleeding at home: Choose an item. …
Actions taken: Click here if you want to enter text.

Treatment - Medicinal
Medication and dosage: Click here if you want to enter text.

7j. Skin phenomena

7j-1. Itch

Current situation: Click here if you want to enter text.

Expectation / "Is it likely that this will occur in this palliative process?":
Click here if you want to enter text.

Goals child/parents: Click here if you want to enter text.

Comments on current situation
Click here if you want to enter text.

Treatment – Non-medicinal
Actions taken: Click here if you want to enter text.

Treatment - Medicinal (Both local and systemic)
Medication and dosage: Clear description of ointments, regularity, quantity, spots. etc.

7j-2. Wounds/decubitus/mucositis

Current situation: Click here if you want to enter text.

Expectation / "Is it likely that this will occur in this palliative process?":
Click here if you want to enter text.

Goals child/parents: Click here if you want to enter text.

Comments on current situation
Click here if you want to enter text.

Diagnostics
Extensive description per location; redness, size, depth and shape, wound edges, maceration, the degree of exudate and bleeding, tendency, odor, swelling, heat and pain. Gradation (stage) per spot!

Treatment
Actions taken: Click here if you want to enter text.

7k. Other

Click here if you want to enter text.

8. Alternative therapies and relaxation/wellness

Overview complementary therapies

Click here if you want to enter text.

Relaxation/wellness actions

Click here if you want to enter text.

9. History of change

Last updated: Choose date

Main changes: Click here if you want to enter text.

10. Other

Click here if you want to enter text.
