# Supplementary material for: The Practical Application of the Individual Care Plan for Pediatric Palliative Care: A Mixed-Method Study
Source: Children (Basel). 2024 Aug 11;11(8):967. doi: 10.3390/children11080967 (PMC11352542; doi:10.3390/children11080967)
Supplement: Supplementary file 1 [file children-11-00967-s001.zip › Supplementary_S2_topic_guide_parents.pdf]

Topic guide parents

Introduction to the research:

- Thanks for participating. Both questionnaire and interview.
- Short explanation about research; cause, aim, phasing.
- Interview lasts 45-60 minutes, will be recorded. Afterwards written down anonymously, video/audio files are deleted.
- If there are any ambiguities after transcribing, or if unclear what was meant when reading back, I will contact you.
- No right or wrong answers, is about how it's been for you
- Any questions?
- Agree we start? Indicate when a break is needed.

Questionnaire focused mainly on lay-out and completeness. This conversation is more about what the ICP has meant in the care for your child, in the collaboration with and between healthcare professionals.

Opening question: tell me about [child].

Would you please go back to the moment when the ICP was first discussed and outline that situation from there until the moment the ICP was finalized; when wat this ins year/disease process, who took the initiative, what was the reason, how did the process proceed.

Theme	Interview question	Any additional questions
Process	- Back to the moment when the ICP was first discussed – outline the situation from there to the moment the ICP was finalized	- Year<br>- At what stage in the illness trajectory?<br>- Was that a logical moment for you?<br>- Who took the initiative?<br>- What was the reason?<br>- What was the goal?<br>- One conversation or multiple?<br>- How did you feel when the ICP was discussed?<br>- How were those conversations for you at that moment?<br>- How do you look back on it now?<br>- Which healthcare professionals were involved?
Input parent/child	- Can you describe your role in completing the ICP?<br>- In what way has consideration been given to what is important for your child and family?	- Have you been involved in the drawing up?<br>- Was the child involved in the drawing up?

		- How do you look back on the involvement of you/your child?<br>- How is this reflected in the ICP?<br>- How were choices/decisions made?<br>- Does the child do something with the ICP themselves?
Use of the ICP	- How do healthcare professionals deal with the ICP?<br>- What do you think of the content of the ICP?<br>- Which part of the ICP do you attach value to?	- In thinking about the future, do they recognize the child and the situation in it<br>- Feasibility<br>- Extent/manner of use<br>- Do you have the impression that it has already been looked at before you enter the conversation?<br>- Situation in which the ICP was not used while you expected it to be<br>- Are there subjects missing? If so, which ones?<br>- Are there parts that do not apply to your child? If so, which parts?
Meaning ICP in daily life	- Could you describe a situation in which the ICP played a role?<br>- What does the ICP mean to you as a parent?<br>- What does the ICP mean for your family?	- How was the ICP perceived by them?<br>- What did it do?<br>- Who did you discuss it with?<br>- Is it present at your home?<br>- Do you ever look into it?<br>- What do you think about it then?<br>- When do you look at it?
