# Supplementary material for: The Practical Application of the Individual Care Plan for Pediatric Palliative Care: A Mixed-Method Study
Source: Children (Basel). 2024 Aug 11;11(8):967. doi: 10.3390/children11080967 (PMC11352542; doi:10.3390/children11080967)
Supplement: Supplementary file 1 [file children-11-00967-s001.zip › Supplementary_S4_questionnaire_parents.pdf]

Welcome

Thank you for participating in the study on improving the Individual Care Plan (ICP).

You were able to read about this study on the website www.izpproject.com. Why we are doing this, what you can expect, and what is expected of you. You have indicated that you would like to participate in the questionnaire. The questionnaire has four sections. The first section consists of background questions about yourself and your child. This information helps us to better understand the answers to the questions in the rest of the questionnaire. The questions after that are about what you think of the content and lay-out of the ICP and about your experiences using the ICP. In each section, you will be given a brief explanation of what is expected of you in that section.

We ask you to answer the questionnaire as honestly and accurately as possible. There are no right or wrong answers. For most questions you can choose from several answers. Sometimes we ask you to explain your answer a little further. Try to write this down as clearly as possible. Do not hesitate to be critical. It helps us to improve the ICP better.

Are you unable to complete the questionnaire in one go? You can stop at any time and close the questionnaire. Remember the code that appears on the screen (or take a picture of it) so that you can open the questionnaire again. You will then automatically start at the question where you left off.

General information

The questionnaire starts here with some background questions about you and your child.

What is your age	….. (in years)
What is your highest completed education	○ No education (elementary school, or primary education not completed)<br>○ Primary education (primary education, special primary education)<br>○ Primary or preparatory vocational educations (such as LTS, LEAO, LHNO, VMBO)<br>○ Secondary general education (such as MAVO, (M)ULO, MBO-short, VMBO-t)<br>○ Intermediate vocational education or vocational accompanying education (such as MBO-long, MTS, MEAO, BOL, BBL INAS)<br>○ Higher general and preparatory scientific education (such as HAVO, VWO, Atheneum, Gymnasium, HBS, MMS)<br>○ Higher vocational education (such as HBO, HTS, HEAO, kandidaats wetenschappelijk onderwijs, bachelors)<br>○ Scientific education (university)<br>○ Other, namely…
I am …. of my child with an individual care plan	○ Parent<br>○ Caretaker<br>○ Other, namely…
My child is a	○ Boy<br>○ Girl<br>○ Other
What is your child's age at this time	……………… (in years. If your child has passed away please indicate that below, other questions will follow for your.)
My child has since passed away	○ Yes
What was your child's age at the time of death?	……………… (in years)
On what date did your child pass away?	…………….
What illness or diagnosis of your child was the reason for drawing up the individual care plan?	…………….
What was the immediate reason for drawing up the individual care plan?	…………….
In what year was the care plan first completed?	…………….
Has there been an update (or multiple updates) to the care plan?	○ No<br>○ Yes
How much time after the drawing up the care plan was this?	…………….
What was your child's estimated life expectancy at the time the individual care plan was being drawn up (in months or years)?	…………….

Content of the ICP

The following questions are about your opinion on the content of the Individual Care Plan (ICP).

We ask you to indicate for each part of the ICP whether you agree with the statements. The statements are about the following 3 points:

1. How important you find the component in the ICP.
Is this a topic about which information and/or agreements should be in the ICP?

2. How complete you find the section.
Does it contain all relevant information? Or are important things missing?

3. How user-friendly do you find the section.
Is it easy to use in everyday practice?

You can indicate your answer on a scale from “completely disagree” (1) to “completely agree” (5). There is also room for comments. Here you can, for example, clarify your answer, indicate why you think something is wrong or leave suggestions. Not sure which part of the care plan it concerns? Below each question you will see an example of the section in question.

**1. Care plan data**	
a. This section of the care plan is important to the ICP<br>b. This section of the care plan is complete<br>c. This part of the care plan is user-friendly	(1) Complete disagree – (5) completely agree
Can you indicate why you disagree with statement 1a?<br>Can you indicate why you disagree with statement 1b?<br>Can you indicate why you disagree with statement 1c?	…………………………………..
Any comments about the section “care plan data”	…………………………………..
**2. General information**	
a. This section of the care plan is important to the ICP<br>b. This section of the care plan is complete<br>c. This part of the care plan is user-friendly	(1) Complete disagree – (5) completely agree
Can you indicate why you disagree with statement 2a?<br>Can you indicate why you disagree with statement 2b?<br>Can you indicate why you disagree with statement 2c?	…………………………………..
Any comments about the section “General information”	…………………………………..
**3. Social map/psychosocial aspects**	
a. This section of the care plan is important to the ICP<br>b. This section of the care plan is complete<br>c. This part of the care plan is user-friendly	(1) Complete disagree – (5) completely agree

Can you indicate why you disagree with statement 3a?<br>Can you indicate why you disagree with statement 3b?<br>Can you indicate why you disagree with statement 3c?	………………………………………..
Any comments about the section “Social map/psychosocial aspects”	………………………………………..
**4. Needs, wishes and goals**	
a. This section of the care plan is important to the ICP<br>b. This section of the care plan is complete<br>c. This part of the care plan is user-friendly	(1) Complete disagree – (5) completely agree
Can you indicate why you disagree with statement 4a?<br>Can you indicate why you disagree with statement 4b?<br>Can you indicate why you disagree with statement 4c?	………………………………………..
Any comments about the section “Needs, wishes and goals”	………………………………………..
**5. Medication including dosage**	
a. This section of the care plan is important to the ICP<br>b. This section of the care plan is complete<br>c. This part of the care plan is user-friendly	(1) Complete disagree – (5) completely agree
Can you indicate why you disagree with statement 5a?<br>Can you indicate why you disagree with statement 5b?<br>Can you indicate why you disagree with statement 5c?	………………………………………..
Any comments about the section “Medication including dosage”	………………………………………..
**6. Nutrition**	
a. This section of the care plan is important to the ICP<br>b. This section of the care plan is complete<br>c. This part of the care plan is user-friendly	(1) Complete disagree – (5) completely agree
Can you indicate why you disagree with statement 6a?<br>Can you indicate why you disagree with statement 6b?<br>Can you indicate why you disagree with statement 6c?	………………………………………..
Any comments about the section “Nutrition”	………………………………………..
**7. Symptomatology**	
a. This section of the care plan is important to the ICP<br>b. This section of the care plan is complete<br>c. This part of the care plan is user-friendly	(1) Complete disagree – (5) completely agree

Can you indicate why you disagree with statement 7a?<br>Can you indicate why you disagree with statement 7b?<br>Can you indicate why you disagree with statement 7c?	……………………………………..
Any comments about the section “Symptomatology”	……………………………………..
**8. Alternative therapies and relaxation/wellness**	
a. This section of the care plan is important to the ICP<br>b. This section of the care plan is complete<br>c. This part of the care plan is user-friendly	(1) Complete disagree – (5) completely agree
Can you indicate why you disagree with statement 8a?<br>Can you indicate why you disagree with statement 8b?<br>Can you indicate why you disagree with statement 8c?	……………………………………..
Any comments about the section “Alternative therapies and relaxation/wellness”	……………………………………..
**9. History of change**	
a. This section of the care plan is important to the ICP<br>b. This section of the care plan is complete<br>c. This part of the care plan is user-friendly	(1) Complete disagree – (5) completely agree
Can you indicate why you disagree with statement 9a?<br>Can you indicate why you disagree with statement 9b?<br>Can you indicate why you disagree with statement 9c?	……………………………………..
Any comments about the section “History of change”	……………………………………..
**10. Other**	
a. This section of the care plan is important to the ICP<br>b. This section of the care plan is complete<br>c. This part of the care plan is user-friendly	(1) Complete disagree – (5) completely agree
Can you indicate why you disagree with statement 10a?<br>Can you indicate why you disagree with statement 10b?<br>Can you indicate why you disagree with statement 10c?	……………………………………..
Any comments about the section “Other”	……………………………………..

Lay-out of the IZP.

The following questions are about your opinion on the lay-out of the Individual Care Plan (ICP).

Currently, the ICP is a fillable Word Document. We know from many parents and healthcare professionals that they do not find this a pleasant working document. For this reason, a fully digital version will be developed in the future, which will be available in a secure environment.

Do you think this change to an online environment will improve the ICP?	○ No<br>○ Yes
Can you indicate why you think so?	…………….
Which of these options do you think is important for an ICP? You can give multiple answers.	☐ The guideline “palliative care for children” should be accessible from the ICP so that I can easily view the recommendations from the guideline<br>☐ Only completed fields should be visible in the final ICP<br>☐ It must be possible to attach a contingency plan (including treatment restrictions) to the OZP when acute death is expected<br>☐ The IZP must be easy to print as a PDF file<br>☐ The IZP must be part of the electronic patient record in the hospital<br>☐ The IZP must be linked to different healthcare systems (also outside the hospital)<br>☐ Everyone involved in the care of a child must have automatic access (thus also healthcare professionals wo are involved once)<br>☐ I have no specific requirements<br>☐ Other, namely….<br>(Note: we feel it is important to include your requirements in the development of the ICP, but cannot guarantee that all wishes will be realized.)
If you have any comments, ideas or suggestions regarding the lay-out of the ICP, please leave them here:	…………..

Experiences with the ICP

The questions that follow are about how the Individual Care Plan (ICP) is drawn up and how it is used by you and the healthcare professionals.

Can you indicate the length of time between your child's illness or diagnosis becoming known an the drawing up of the ICP?	○ 0-2 months<br>○ 3-6 months<br>○ 7-12 months<br>○ Longer than a year, but no longer than two years<br>○ Longer than two years
Can you indicate the reason this period was longer than 2 months?	………………
Did you know who was responsible for drawing up the IZP?	○ No<br>○ Yes
Wat is the position of the person who was responsible?	…………………<br>(for example pediatrician or nurse)
In what way was the drawing up of the ICP done?<br>- Did the healthcare professional talk with you about the information and agreements recorded in the ICP (this may have been in one or more conversations)?<br>- Was the ICP drawn up by the healthcare professional during the conversations with you?<br>- Was the ICP drawn up by the healthcare professionals outside of the conversations with you?<br>- Was it possible to suggest modifications to the information and agreements made in the draft version(s) of the IZP?<br>- Was it possible to suggest adjustments once the ICP was finalized?<br>- Was your child involved in the drawing up of the ICP?	○ No<br>○ Yes<br>○ I don't know
Can you indicate why your child was not involved?	○ This was not possible due to a developmental delay, communication issues or too young age of my child<br>○ Other, namely…
You indicate that the ICP was drawn up by the healthcare professional during the conversation with you. How did you experience that?	…………………
You indicate that the ICP was drawn up by the healthcare professional outside of the conversation with you. How did you reel about that?	…………………
Please indicate the extent to which you agree with the following statements	(1) Complete disagree – (5) completely agree

- The ICP allows me to better discuss my child's care and treatment with healthcare professionals<br>- Because of the IZP, my child's healthcare professionals work better together<br>- Healthcare professionals at the hospital know the content of the ICP<br>- Healthcare professionals outside the hospital know the content of the ICP<br>- The agreements in the ICP fit what I think is important for my child and my family<br>- The goals of care and treatment as written down in the ICP fit what I think is important for my child and my family<br>- - The ICP helps me as a parent/caregiver to have more control in the care of my child	
Any comments on the preceding statements	………………
Imagine if your child did not have a ICP. Would the care then worse, about the same or better?	○ A lot worse<br>○ Worse<br>○ About the same<br>○ Better<br>○ Way better<br>○ I don't know
What do you like most about having an IZP	……………
Is there anything you would like to change about the way the ICP is used?	○ No<br>○ Yes
What would you like to change?	…………….
How likely would you be to recommend the ICP to other parents of a child with a serious condition?	○ Not likely at all<br>○ Not likely<br>○ Neither unlikely nor likely<br>○ Likely<br>○ Most likely
