# Supplementary material for: The Practical Application of the Individual Care Plan for Pediatric Palliative Care: A Mixed-Method Study
Source: Children (Basel). 2024 Aug 11;11(8):967. doi: 10.3390/children11080967 (PMC11352542; doi:10.3390/children11080967)
Supplement: Supplementary file 1 [file children-11-00967-s001.zip › Supplementary_S5_questionnaire_healthcare_professionals.pdf]

Welcome

Thank you for participating in the survey "The next step in the child and family-centered individual care plan".

This questionnaire is part of the research project you have been informed about on the website www.izpproject.com. With this research we want to map the experiences of children, parents and healthcare professionals with the Individual Care Plan (ICP).

The questionnaire consists of 5 parts. The first section consists of background questions about yourself, your position, your experiences with pediatric palliative care and your role regarding the Individual Care Plan (ICP). We need this information to understand the answers to the subsequent questions. The questions in parts 2 through 4 are about the content and lay-out of the IZP and your experience using the ICP. The last section deals with factors that (may) affect the use of the IZP.

Prior to each section, you will receive a brief explanation of what is expected of you in that section.

We ask that you answer the questionnaire as honestly and accurately as possible. There are no "right" or "wrong" answers.

Most questions are multiple choice. Some questions have space for comments. Please try to express your comments as clearly and in as much detail as possible. Do not hesitate to be critical.

Are you unable to complete the questionnaire in one go? You can stop at any time and close the questionnaire. Remember the code that appears on the screen so that you can open the questionnaire again. You will then automatically start at the question where you left off.

General information

The questionnaire starts with some background questions about yourself, your position, your experience with pediatric palliative care and your role regarding the Individual Care Plan (ICP).

What is your age?	………..
What is your gender?	○ Man<br>○ Woman<br>○ Other, namely….
What is your highest completed education	○ No education (elementary school, or primary education not completed)<br>○ Primary education (primary education, special primary education)<br>○ Primary or preparatory vocational educations (such as LTS, LEAO, LHNO, VMBO)<br>○ Secondary general education (such as MAVO, (M)ULO, MBO-short, VMBO-t)<br>○ Intermediate vocational education or vocational accompanying education (such as MBO-long, MTS, MEAO, BOL, BBL INAS)<br>○ Higher general and preparatory scientific education (such as HAVO, VWO, Atheneum, Gymnasium, HBS, MMS)<br>○ Higher vocational education (such as HBO, HTS, HEAO, kandidaats wetenschappelijk onderwijs, bachelors)<br>○ Scientific education (university)<br>Other, namely…
At which institution/organization are you employed?	………….
What is your position?	○ Pediatrician<br>○ Pediatrician subspecialist<br>○ Residency in pediatrics<br>○ Physician in intellectual disability care<br>○ Specialized nurse (including CPC nurse)<br>○ Pediatric hospital nurse<br>○ Pediatric home care nurse<br>○ Pediatric nurse in pediatric hospice<br>○ Spiritual care provider<br>○ Psychologist<br>○ Social worker<br>○ Pedagogical assistant<br>○ General practitioner<br>○ Other, namely….
How long have you worked in your current position?	………………. (Indicated if it is in years or months)
How long have you worked in the care for seriously ill children with a life-threatening or life-limiting condition?	………………. (Indicated if it is in years or months)

Have you had any schooling in pediatric palliative care (for example, training or E-learning)?	○ No<br>○ Yes
Can you indicate what kind of training you have followed?	………..
What role do you generally have with regard to the ICP?	○ I am responsible for the filling in/drawing up of the ICP (and working with it)<br>○ I work with the ICP, but do not fill in the ICP myself
Can you indicate the main reason why you do not fill in/draw up the ICP yourself?	○ Filling in/drawing up an ICP does not suit my position<br>○ I do not feel competent in filling in/drawing up an ICP<br>○ There has not been a situation in which I could have taken on the task of filling in/drawing up an ICP<br>○ Other, namely…
How many ICP's have you drawn up?	○ 1 ICP<br>○ 2-5 ICP's<br>○ 6-10 ICP's<br>○ More than 10 ICP's
Do you use a modified version of the ICP in your institution?	○ No<br>○ Yes
With how many ICP's have you worked?	○ 1 ICP<br>○ 2-5 ICP's<br>○ 6-10 ICP's<br>○ More than 10 ICP's
If you had to give a mark for your worst experience in drawing up or working with an Individual Care Plan, what would you give?	……………….
If you had to give a mark for your best experience in drawing up or working with an Individual Care Plan, what would you give?	……………..
If you do not work at a University Medical Center: how was the cooperation with the Children's Palliative Care Teams or Children's Palliative Care Networks?	…………………….

Content of the IZP

The following questions are about your opinion on the contents of the Individual Care Plan (ICP). If you have dealt with (drawn up or worked with) multiple ICPs, answer the questions for your average experience. You will be asked to indicate for each part of the ICP whether you agree with the statements. These statements are about the following 3 points:

1. How important you find the component in the ICP.
Is this a topic about which information and/or agreements should be in the ICP?

2. How complete you find the section.
Does it contain all relevant information? Or are important things missing?

3. How user-friendly do you find the section.
Is it easy to use in everyday practice?

You can indicate your answer on a scale from “completely disagree” (1) to “completely agree” (5). There is also room for comments. Here you can, for example, clarify your answer, indicate why you think something is wrong or leave suggestions. Not sure which part of the care plan it concerns? Below each question you will see an example of the section in question.

**1. Care plan data**	
a. This section of the care plan is important to the ICP<br>b. This section of the care plan is complete<br>c. This part of the care plan is user-friendly	(1) Complete disagree – (5) completely agree
Can you indicate why you disagree with statement 1a?<br>Can you indicate why you disagree with statement 1b?<br>Can you indicate why you disagree with statement 1c?	…………………………………….
Any comments about the section “care plan data”	…………………………………….
**2. General information**	
a. This section of the care plan is important to the ICP<br>b. This section of the care plan is complete<br>c. This part of the care plan is user-friendly	(1) Complete disagree – (5) completely agree
Can you indicate why you disagree with statement 2a?<br>Can you indicate why you disagree with statement 2b?<br>Can you indicate why you disagree with statement 2c?	…………………………………….
Any comments about the section “General information”	…………………………………….
**3. Social map/psychosocial aspects**	
a. This section of the care plan is important to the ICP<br>b. This section of the care plan is complete	(1) Complete disagree – (5) completely agree

c. This part of the care plan is user-friendly	
Can you indicate why you disagree with statement 3a?<br>Can you indicate why you disagree with statement 3b?<br>Can you indicate why you disagree with statement 3c?	………………………………………..
Any comments about the section “Social map/psychosocial aspects”	………………………………………..
**4. Needs, wishes and goals**	
a. This section of the care plan is important to the ICP<br>b. This section of the care plan is complete<br>c. This part of the care plan is user-friendly	(1) Complete disagree – (5) completely agree
Can you indicate why you disagree with statement 4a?<br>Can you indicate why you disagree with statement 4b?<br>Can you indicate why you disagree with statement 4c?	………………………………………..
Any comments about the section “Needs, wishes and goals”	………………………………………..
**5. Medication including dosage**	
a. This section of the care plan is important to the ICP<br>b. This section of the care plan is complete<br>c. This part of the care plan is user-friendly	(1) Complete disagree – (5) completely agree
Can you indicate why you disagree with statement 5a?<br>Can you indicate why you disagree with statement 5b?<br>Can you indicate why you disagree with statement 5c?	………………………………………..
Any comments about the section “Medication including dosage”	………………………………………..
**6. Nutrition**	
a. This section of the care plan is important to the ICP<br>b. This section of the care plan is complete<br>c. This part of the care plan is user-friendly	(1) Complete disagree – (5) completely agree
Can you indicate why you disagree with statement 6a?<br>Can you indicate why you disagree with statement 6b?<br>Can you indicate why you disagree with statement 6c?	………………………………………..
Any comments about the section “Nutrition”	………………………………………..
**7. Symptomatology**	
a. This section of the care plan is important to the ICP<br>b. This section of the care plan is complete	(1) Complete disagree – (5) completely agree

c. This part of the care plan is user-friendly	
Can you indicate why you disagree with statement 7a?<br>Can you indicate why you disagree with statement 7b?<br>Can you indicate why you disagree with statement 7c?	………………………………………..
Any comments about the section "Symptomatology"	………………………………………..
**8. Alternative therapies and relaxation/wellness**	
a. This section of the care plan is important to the ICP<br>b. This section of the care plan is complete<br>c. This part of the care plan is user-friendly	(1) Complete disagree – (5) completely agree
Can you indicate why you disagree with statement 8a?<br>Can you indicate why you disagree with statement 8b?<br>Can you indicate why you disagree with statement 8c?	………………………………………..
Any comments about the section "Alternative therapies and relaxation/wellness"	………………………………………..
**9. History of change**	
a. This section of the care plan is important to the ICP<br>b. This section of the care plan is complete<br>c. This part of the care plan is user-friendly	(1) Complete disagree – (5) completely agree
Can you indicate why you disagree with statement 9a?<br>Can you indicate why you disagree with statement 9b?<br>Can you indicate why you disagree with statement 9c?	………………………………………..
Any comments about the section "History of change"	………………………………………..
**10. Other**	
a. This section of the care plan is important to the ICP<br>b. This section of the care plan is complete<br>c. This part of the care plan is user-friendly	(1) Complete disagree – (5) completely agree
Can you indicate why you disagree with statement 10a?<br>Can you indicate why you disagree with statement 10b?<br>Can you indicate why you disagree with statement 10c?	………………………………………..
Any comments about the section "Other"	………………………………………..
Is there something you miss in the current ICP?	………………………………………..

Lay-out of the ICP

The following questions are about your opinion on the lay-out of the Individual Care Plan (ICP).

At the moment the ICP is a fillable Word document. We know from healthcare professionals and parents that they do not find this document pleasant to work with. For this reason, a fully digital version will be developed in the future, which will be available in a secure environment.

Do you think this will improve the ICP?	○ No<br>○ Yes
Can you indicate why you think so?	…………….
What other changes would improve the current ICP according to you?	…………….
What would you like to keep in the renewed ICP?	……………..
Which of the following adjustments would you like to see implemented in the renewed ICP? It is possible to give multiple answers.	☐ The guideline “palliative care for children” should be accessible from the ICP so that I can easily view the recommendations from the guideline<br>☐ Only completed fields should be visible in the final ICP<br>☐ It must be possible to attach a contingency plan (including treatment restrictions) to the OZP when acute death is expected<br>☐ The IZP must be easy to print as a PDF file<br>☐ The IZP must be part of the electronic patient record in the hospital<br>☐ The IZP must be linked to different healthcare systems (also outside the hospital)<br>☐ Everyone involved in the care of a child must have automatic access (thus also healthcare professionals wo are involved once)<br>☐ I have no specific requirements<br>☐ Other, namely….<br>(Note: we feel it is important to include your requirements in the development of the ICP, but cannot guarantee that all wishes will be realized.)
If you have any comments, ideas or suggestions regarding the lay-out of the ICP, please leave them here:	……………

Experiences with the ICP

The questions that follow now concern the drawing up of the individual Care Plan (ICP) and the way in which it is used in practice. If you have dealt with (drawn up or worked with) multiple ICPs, please answer the questions for the most recent case you have dealt with.

How long was the period between diagnose of the disease that was the reason for an ICP and the drawing up of an ICP?	○ 0-2 months<br>○ 3-6 months<br>○ 7-12 months<br>○ Longer than a year, but no longer than two years<br>○ Longer than two years
What do you think was the reason that this period was longer than 2 months?	…………….
Were you the child's chief practitioner	○ No<br>○ Yes
Who do you think should draw up the ICP?	…………..
Did parents/caregiver know who was responsible for drawing up the ICP?	○ No<br>○ Yes<br>○ I don't know
Can you indicate why they did not know this?	…………..
Who do you think is responsible for the ICP?	○ Parents<br>○ Chief practitioner<br>○ The one who drew up the ICP<br>○ Other, namely….
How was the ICP drawn up?<br>- Have you spoken with parents/carers about which information and agreements should be recorded in the ICP? (this may have been in one or more conversations)<br>- Did you draw up the ICP during the conversations with parents/carers?<br>- Did you draw up the ICP outside the conversations with parents/carers?<br>- Were parents/carers able to provide feedback on the established information and agreements in the draft version(s) of the ICP?<br>- Were parents/carers able to propose changes to the recorded information an agreements in the ICP after the ICP had been finalized?<br>- Was the child involved in drawing up the ICP?	○ No<br>○ Yes
Can you indicate why your child was not involved?	○ This was not possible due to a developmental delay, communication issues or too young age of my child<br>○ Other, namely…
You indicate that you drew up the ICP during the conversation with parents/carers. How did you experience that?	…………………..

You indicate that you drew up the ICP outside of the conversation with parents/carers. How did you reel about that?	……………………
Any comments on the above questions	…………………
How was the ICP drawn up?<br>- Has your colleague spoken with parents/carers about which information and agreements should be recorded in the ICP? (this may have been in one or more conversations)<br>- Did your colleague draw up the ICP during the conversations with parents/carers?<br>- Did your colleague draw up the ICP outside the conversations with parents/carers?<br>- Were parents/carers able to provide feedback on the established information and agreements in the draft version(s) of the ICP?<br>- Were parents/carers able to propose changes to the recorded information an agreements in the ICP after the ICP had been finalized?<br>- Was the child involved in drawing up the ICP?	○ No<br>Yes
Can you indicate why your child was not involved?	○ This was not possible due to a developmental delay, communication issues or too young age of my child<br>○ Other, namely…
Please indicate the extent to which you agree with the following statements<br>- The ICP supports me in discussing care and treatment with parents/carers.<br>- The IZP supports me in discussing care and treatment with the child itself.<br>- The ICP contains all the elements necessary for collaboration between healthcare professionals who are involved with the child and the family.<br>- Healthcare professionals in the hospital are aware of the content of the ICP.<br>- Healthcare professionals outside the hospital are aware of the content of the ICP.<br>- The agreements in the ICP are in line with what the child and parents/carers consider important for themselves and the family.<br>- The ICP provides a good representation of the goals that I consider important as	○ (1) Complete disagree – (5) completely agree

a healthcare professional in the care ant treatment of children and families.<br>- The ICP helps parents/cares to have more control over the care for their child.	
You have indicated that the ICP does not contain all the elements necessary for collaboration between healthcare professionals who are involved with the child and family. Can you indicate which elements are missing?	…………….
Any comments on the above questions	………………..
Who do you think should have access to the ICP?	………………..
Imagine if there was no ICP. Would the care for children with a serious condition be worse, about the same or better?	○ A lot worse<br>○ Worse<br>○ About the same<br>○ Better<br>○ Way better<br>○ I don't know
What do you like the most about working with an ICP?	…………….
Is there anything you would like to change about the way the ICP is used?	○ No<br>○ Yes
What would you like to change?	……………….
How likely is it that you would recommend the ICP to parents?	○ Not likely at all<br>○ Not likely<br>○ Neither unlikely nor likely<br>○ Likely<br>○ Most likely
How likely is it that you would recommend the ICP to colleagues?	○ Not likely at all<br>○ Not likely<br>○ Neither unlikely nor likely<br>○ Likely<br>○ Most likely
Has it happened in the past two years that a patient was eligible for an ICP, but did not have an ICP?	○ No<br>○ Yes
Can you indicate the reason for this?	…………
Which children do you think qualify for an ICP?	…………
The "Zakboekje IZP" is an accompanying manual of the ICP and is intended as a writing aid when drawing up the ICP. Are you familiar with that Zakboekje?	○ No<br>○ Yes
Do you use the Zakboekje?	○ Every time I draw up an ICP<br>○ Sometimes I do sometimes I don't<br>○ I never use it<br>○ Other, namely…
Can you indicate why you do not use the Zakboekje?	………….
How do you use the Zakboekje? (possible to fill in multiple options)	☐ As a guideline when completing all parts of the ICP

	☐ As a guideline when completing specific parts of the ICP<br>☐ As a reference in general<br>☐ Other, namely..
Which part or parts are they?	…………..
Can you indicate what is the most important reason for you to use the Zakboekje?	…………..

The ICP as an innovation

The next part of the questionnaire is about factors that (may) influence the use of the Individual Care Plan (ICP). If you have dealt with multiple ICPs (drawn up or worked with), we ask you to bear in mind your general experience with the ICP when answering the statements.

When patients are mentioned, this means: children or young people with a life-threatening or life-limiting condition.

The questions from the Measurement Instrument for Determinants of Innovations (MIDI) (12) were presented to healthcare professionals.
